# Sequestering survival: sponge-like proteins in phage evasion of bacterial immune defenses

**DOI:** 10.3389/fimmu.2025.1545308

**Published:** 2025-04-17

**Authors:** Lan Wang, Ruoqi Zheng, Leiliang Zhang

**Affiliations:** ^1^ Department of Clinical Laboratory Medicine, The First Affiliated Hospital of Shandong First Medical University & Shandong Provincial Qianfoshan Hospital, Jinan, Shandong, China; ^2^ Department of Pathogen Biology, School of Clinical and Basic Medical Sciences, Shandong First Medical University & Shandong Academy of Medical Sciences, Jinan, Shandong, China

**Keywords:** sponge-like proteins, cyclic oligonucleotides, phage, CBASS, Thoeris

## Abstract

By executing abortive infection, bacterial immune defense systems recognize phage components and initiate the production of various second messengers that target specific downstream effectors responsible for nucleic acid degradation, membrane destruction, or metabolite depletion. Notably, the sponge-like proteins encoded by phages, such as Tad1, Tad2, and Acb2, can inhibit abortive infection by sequestering, rather than degrading, these bacterial second messengers. This interference disrupts the activation of the effectors involved in the immune response. Most significantly, sponge-like proteins can simultaneously encapsulate diverse signals, effectively preventing the cell suicide mechanisms triggered by different bacterial immune systems, such as the cyclic nucleotide-based antiphage signaling system (CBASS) and Thoeris. The discovery of these sponge-like proteins reveals a remarkable strategy for suppressing innate immunity, ensuring viral replication and propagation. This greatly enhances our understanding of the ongoing arms race between hosts and viruses.

## 1 Introduction

The immune defense system serves as an inherent barrier to block pathogen invasion and maintain biological homeostasis. In addition to the well-known human immune defense system, bacteria also equip themselves with powerful immune mechanisms to defend against their natural viral enemies, phages. Intriguingly, some bacterial immune systems are triggered by the recognition of specific viral components and initiate a self-destruct process. This process generates distinct cyclic oligonucleotides (cOs) that activate downstream effectors, which carry out suicide instructions to prevent viral replication. The mode of suicide execution depends on the type of effector involved, which may include RNA or DNA degradation, membrane destruction, and metabolite depletion (1). For instance, both the type III clustered regularly interspaced palindromic repeats (CRISPR)-CRISPR associated protein (Cas) system and the cyclic nucleotide-based anti-phage signaling system (CBASS) utilize the endonuclease NucC to induce cell suicide. They rely on cA3 as the second messenger produced by the Cas10 subunit of the VmeCmr complex and CD-NTase, respectively, for the activation of NucC (2, 3). Additionally, the pyrimidine cyclase system for anti-phage resistance (PYCSAR) activates a TIR-domain effector for NAD+ depletion by generating cCMP or cUMP, while the Thoeris (the Egyptian protective deity of childbirth and fertility) synthesizes the cADPR isomer (4, 5). Moreover, various bacterial immune systems employ unique methods for phage recognition (2, 6–8). It is also essential to acknowledge that the interactions and interconnectedness of these systems should not be overlooked.

Faced with such dire survival conditions, phages have evolved effective strategies for immune evasion. One remarkable weapon encoded by phages is the sponge-like protein, which can sequester various signals and thus interfere with the abortive infection process, allowing for the coexistence of bacteria and phages and facilitating phage replication. Currently known sponge-like proteins include anti-CBASS 2 (Acb2), Thoeris anti-defense 1 (Tad1), and Tad2, which exhibit resistance to CBASS or Thoeris, suggesting that phages encoding sponge-like proteins can simultaneously counteract different bacterial immune systems (9, 10).

## 2 Bacterial abortive infection inhibits phage replication

**Figure 1:**
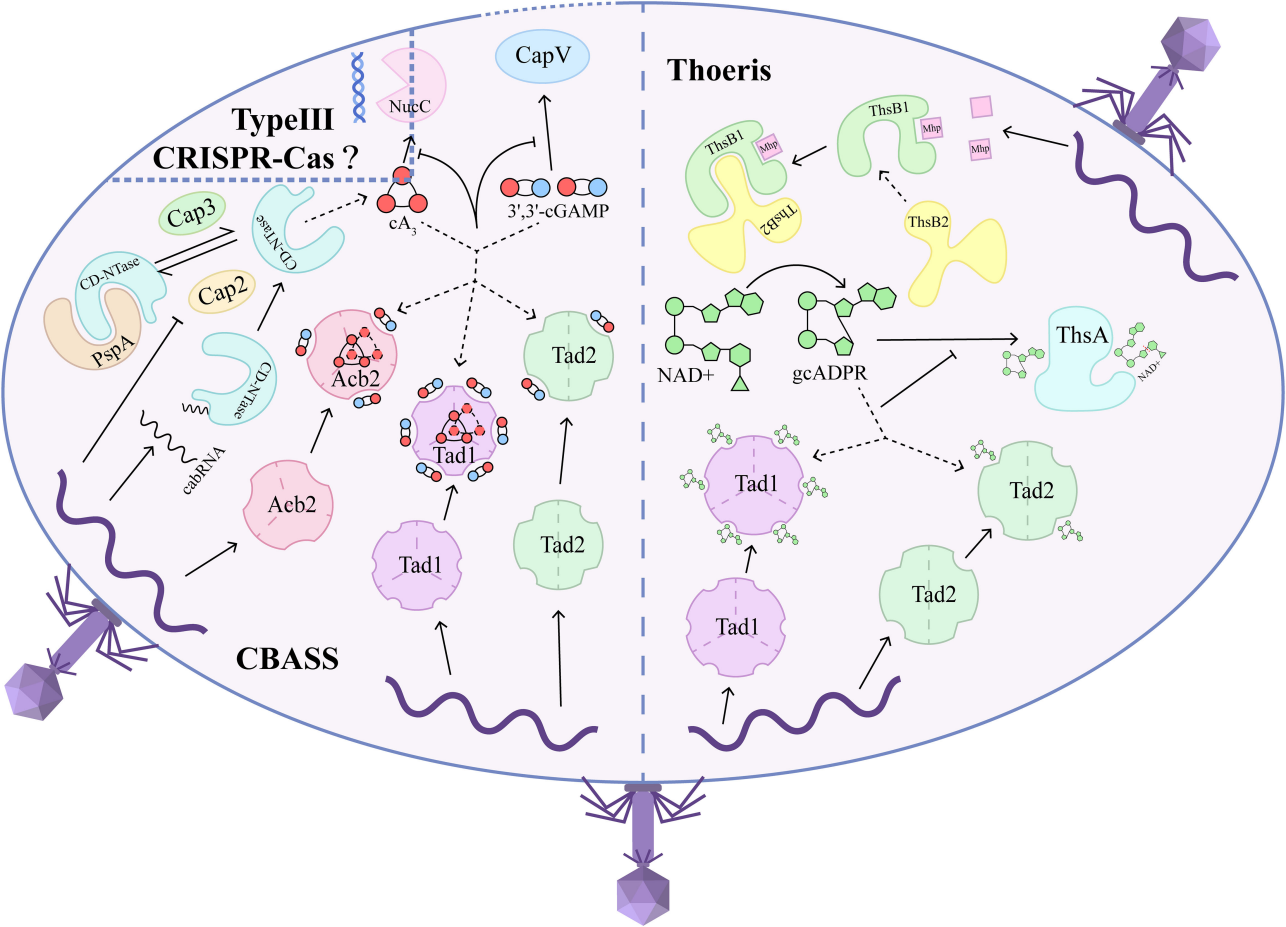
Sponge-like proteins from phages mitigate abortive infection by sequestering second messengers across bacterial immune systems. When phages adhere to bacteria and introduce their genome into the bacterial cytoplasm, systems like CBASS and Thoeris recognize the phage components and generate specific second messengers. They rely on their unique phage sensors, CD-NTase and ThsB, respectively, to activate downstream effectors that execute abortive infection through DNA degradation, membrane destruction, or metabolite depletion. CD-NTase, in conjunction with PspA, stabilizes an inactive state in a reversible manner, similar to ubiquitin modifications, through Cap2 and Cap3 in the absence of phage invasion. When a viral invasion occurs, this cycle is disrupted, liberating CD-NTase and priming its activation to synthesize 3’,3’-cGAMP, cA3, or other cOs that activate various downstream effectors. Notably, CD-NTase can also be initiated by binding to cabRNA transcribed from the phage genome. On the other hand, ThsB1 functions as a phage component sensor that recognizes the Mhp and subsequently recruits ThsB2, which transforms NAD+ into gcADPR, leading to the depletion of NAD+ and ultimately causing cell death. In response, phages express sponge-like proteins to counter abortive infection by sequestering these second messengers, thereby preventing the effectors from executing cell suicide. The sponge-like proteins that have been identified include Acb2, Tad1, and Tad2. To inhibit CBASS, Acb2 and Tad1 can absorb CDNs and CTNs such as 3’,3’-cGAMP and cA3, while Tad2 is specific to CDNs. Additionally, Tad1 and Tad2 can sequester gcADPR to terminate the NAD+ depletion process orchestrated by Thoeris. Type III CRISPR-Cas may be influenced by the shared cA3 signal and NucC effector with CBASS. Consequently, sponge-like proteins can simultaneously defend against multiple immune systems.

Abortive infection is a bacterial immune system strategy for confronting phages, induced by mechanisms such as CBASS, Thoeris, and other immune systems with distinct functions (11). CBASS initiates cell suicide through a variety of downstream effectors, including DNA nucleases, phospholipases, membrane-disrupting proteins, TIR-domain proteins, and immune ATP-nucleosidase domain proteins, which are activated by specific cOs generated by CD-NTase (cGAS/DncV-like nucleotidyltransferase) (11, 12). NucC, activated by cA3, Cap5, activated by 3’2’-cGAMP, and Cap4, activated by cA3 or cAAG, adopt distinct conformations for DNA degradation. Activated NucC forms a hexamer, while activated Cap5 exists as a tetramer, both of which differ significantly from the higher-order complex of activated Cap4, a common oligomerization state of CBASS effectors (3, 13–16). To induce membrane disruption, CapV, activated by 3’3’-cGAMP, targets phosphatidylethanolamine (PE) and phosphatidylglycerol (PG) in the cell membrane, while the higher-order complex of Cap15, activated by CDNs, disrupts the cell membrane (17, 18). Additionally, TIR-SAVED, activated by cA3, and TIR-STING, activated by c-di-GMP, assemble into higher-order filaments that facilitate rapid NAD+ depletion, dependent on the NADase activity of the TIR domain (19–21). Recently discovered ATP nucleosidase, Cap17, a CBASS effector, has been mobilized to defend against phage infection, leading to the degradation of ATP and dATP (12). The innate immunity mediated by CBASS is both inclusive and profound, providing numerous insights for exploring the mechanisms of innate immunity in eukaryotes. Recent studies have illuminated how CD-NTase recognizes characteristic phage components. To activate CBASS, a bacteriophage structural RNA known as cabRNA (CBASS-activating bacteriophage RNA) binds to the positively charged surface of CD-NTase, initiating the generation of second messengers. Alternatively, the complex formed by phage shock protein A (PspA) and CD-NTase, linked by Cap2 (CD-NTase-associated protein 2) in an ubiquitin-like manner, is released upon phage infection to prime the activation of CD-NTase (**Figure 1**) (6, 7).

The Thoeris system comprises ThsA and ThsB, where ThsB1 forms a complex with the phage major head protein (Mhp) to recruit and bind to ThsB2, which synthesizes gcADPR (8, 22). ThsA, consisting of the Smf/DprA-LOG (SLOG) domain, recognizes 1’,3’-gcADPR, while the silent information regulator 2 (SIR2) domain executes NAD+ hydrolysis, driving cell suicide.

Other immune systems, such as PYCSAR, possess cyclases that generate cCMP or cUMP as second messengers to activate TIR-domain effectors that degrade NAD+ (4). Since both TIR domains and CD-NTase are critical proteins that recognize viral components to promote immune reactions in both prokaryotic and eukaryotic cells, CBASS, Thoeris, and other bacterial immune systems play significant roles in the further exploitation of innate immune responses, strengthening the natural barrier against viral invasion.

Conversely, phages have evolved a variety of tactics aimed at circumventing these defenses to ensure successful replication. Many of proteins encoded by phages target these second messengers, effectively halting the programmed cell suicides initiated by bacterial immune responses.

## 3 Sponge-like proteins antagonize bacterial safeguards

The unique abilities of different phages can be attributed to the diversity of their genes. Leavitt et al. and Yirmiya et al. conducted screenings of groups of closely related phages to determine which ones could effectively antagonize Thoeris systems and replicate successfully in *Bacillus subtilis* expressing the Thoeris system (23, 24). As anticipated, specific phages that evaded the Thoeris system were identified, including SBSphiJ7, SPO1, and SPO1L3, which differ from their sensitive relatives within the same family. These phages were first distinguished by contrasting them with other members of their family. Subsequently, they were cloned to identify which gene expressions could help Thoeris-sensitive phages overcome Thoeris-expressing cells (23, 24). From this analysis, *tad1* from SBSphiJ7 and *tad2* from SPO1 and SPO1L3 were selected among the unique genes identified. Phages naturally carrying *tad1* and *tad2*, or those engineered to include these genes, demonstrated the ability to inhibit Thoeris, while those lacking them failed to replicate effectively. This further validated Tad1 and Tad2 as anti-Thoeris proteins.

Additionally, Huiting et al. discovered an extended *orf24* through whole-genome sequencing of phage PaMx41 mutants, leading to the expression of a lengthened gp24 protein consisting of 94 amino acids (25). Homologs of this protein were also identified in PaMx33, PaMx35, and PaMx43, which naturally exhibit anti-CBASS abilities. Further experimental evidence demonstrated that the long gp24 increased the titer of the PaMx41 wild-type phage compared to the short gp24, while phages with depleted orf24 lost their anti-CBASS capability. Remarkably, the addition of long gp24 restored this ability, confirming it as the Acb2 protein (25).

Phylogenetic analysis of Tad1 and Tad2 homologs revealed that they are encoded in the *Myoviridae*, *Podoviridae*, and *Siphoviridae* phage families, as well as in the genomes of prophages (23, 24). According to the phylogenetic tree, Acb2 is encoded in the genomes of 239 tailed phages, which belong to phage families that include *Escherichia*, *Enterobacteria*, *Klebsiella*, *Pseudomonas, Shigella*, *Vibrio*, *Yersinia*, and others. Notably, Acb2 is active during lysogenic induction rather than during lysogenic maintenance (25). Homology studies further indicate that Tad1, Tad2, and Acb2 are present in a variety of phages (23–25), suggesting that these proteins function as common defenders against bacterial immune systems.

## 4 Sponge-like proteins sequester diverse signals rather than degrade them

Unlike proteins with enzymatic activity, sponge-like proteins eliminate second messengers in a concentration-dependent manner rather than relying on a time-dependent process. This means that the higher the concentration of sponge-like proteins, the more molecules they can sequester. This contrasts with enzymes, which degrade specific substrates over time as long as they remain active under appropriate conditions. Moreover, the denaturation of these proteins can restore the activity of various signals that initiate downstream effectors (23–25). The term “sponge” metaphorically reflects the multi-hollow structure of these proteins.

Visually, sponge-like proteins are composed of protomers interconnected by robust chemical bonds, creating pockets for sequestering cOs. Surprisingly, Tad1, Tad2, and Acb2 each possess unique structures and recognition mechanisms for separating specific signals. A previous study found that Tad1 is a dimer interlocked by a wedge-shaped architecture formed by two protomers that include an N-terminal anti-parallel β-sheet (β1-β4) and two long C-terminal helices (α1-α2). This structure differs from the Acb2 protomer, which consists of one short N-terminal helix and two long anti-parallel helices (23, 25). Interestingly, Tad1 has been identified as a hexamer composed of three dimers, and each dimer can sequester two gcADPR molecules or CDNs in corresponding ligand-binding pockets surrounded by four highly conserved loops and the C-terminal tail of a protomer, along with the α1-α2 helices of the other protomer (10, 23). In contrast, Acb2 can only capture three 3’3’-cGAMP molecules, linked by π-π stacking and hydrogen bonds within pockets formed by the N- and C-terminal helices and loops of two protomers arranged in a head-to-head orientation (25). Additionally, the trimer interface of Acb2 forms a channel in the center of the hexamer, binding two CTNs at its ends, which is similar to that of Tad1 (9, 25).

Tad2, another sponge-like protein, is a homotetramer formed by V-shaped dimers at helix α2 and sheet β2, which then interlock along helix α1 to form a complete tetramer. Tad2 sequesters two gcADPR molecules linked by extensive van der Waals interactions and polar interactions in two pockets formed by different protomers of distinct dimers. These pockets are surrounded by loop β1–β2, loop β3–β4, and strand β4 of one protomer, as well as loop β1–β2, loop β4–α3, and helix α3 of another protomer (24). However, the contact points between Tad2 and 1’-3’gcADPR are fewer than those of Tad1 (24), possibly indicating that sponge-like proteins like Tad1 may possess a stronger antiviral capacity than Tad2 under certain circumstances. In contrast to the shared pockets that catch gcADPR and CDNs in Tad1, Tad2 binds two CDNs through an insertion between β2 and β5, independent of the gcADPR pockets (10). CTNs do not bind with Tad2, which suggests a potential necessity for the hexameric structure or the formation of a central channel for cA3 sequestration (10).

Overall, sponge-like proteins represent a credible strategy for countering bacterial immune systems, leveraging their highly efficient expression and capability to directly sequester signals. This promotes prolonged coexistence between bacteria and phages.

## 5 Sponge-like proteins suppress different defense systems simultaneously

The potent functions of sponge-like proteins are vividly illustrated by their ability to bind diverse cOs that mediate various bacterial immune systems, allowing these proteins to suppress multiple defense systems simultaneously (**Figure 1**). Recent studies show that Tad1 can bind to multiple cyclic trinucleotides (CTNs) at two interfaces of trimers formed by three dimers, independently of the pockets used for gcADPR binding. Furthermore, Tad1 is also able to bind cyclic dinucleotides (CDNs) within the same six pockets designated for gcADPR. Specifically, Tad1 exhibits weak binding to 3’3’-cGAMP, cGG, cUA, and cAA, while binding to 3’2’-cGAMP, 2’3’-cGAMP, cA3, and cAAG. Tad1 has the ability to sequester gcADPR, CDNs, and CTNs via independent binding pockets, thereby preventing the simultaneous activation of downstream effectors across different immune systems. Interestingly, Tad1 homologs are conserved in their ability to bind to CTNs and gcADPR but exhibit a broader spectrum of binding to CDNs. Tad1, which has a high affinity for 3’, 3’-cGAMP or 3’3’3’-cyclic AMP-AMP-AMP (cA3), successfully blocks CapV from executing membrane destruction leading to cell death, as well as inhibiting NucC from degrading bacterial DNA, achieving an effectiveness comparable to that of Acb2 (**Figure 1**). Notably, Tad1 is naturally present in phages that are resistant to CBASS and is encoded by *orf184*, suggesting that sponge-like proteins can flexibly inhibit the immune systems of preferred bacterial hosts (10).

CDNs such as 3’2’-cGAMP, 3’3’-cGAMP, cGG, and cUG exhibit strong binding to Tad2, whereas 2’3’-cGAMP binds to Tad2 very weakly. Tad2, which has a high affinity for cGG due to a long insertion between β2 and β5, effectively suppresses TIR-STING signaling, thereby depleting NAD+ and triggering cellular apoptosis. However, because Tad2 shares the same pockets for CDNs, its ability to sequester 3’,3’-cGAMP—mediating membrane destruction in *Pseudomonas aeruginosa* with two distinct CBASS systems—can be compromised when these pockets are saturated by cGG (10).

Previous studies identified Acb2 as a sponge-like protein that, in addition to binding to 3’,3’-cGAMP, can also bind to 2’,3’-cGAMP and 3’,3-cUU/UA/UG/AA with high affinity (25). Further investigations revealed that Acb2 binds to CTNs including cA3 and 3’3’3’-cyclic AMP-AMP-GMP (cAAG), with a higher affinity than that for CDNs, and CDNs and CTNs binding pockets are independent of one another (**Figure 1**) (9). Acb2’s high affinity for cA3 also enables it to inhibit NucC (an endonuclease activated by cA3) from degrading DNA (3, 9). More importantly, Acb2 can sequester both 3’,2’-cGAMP and 2’,3’-cGAMP, which are involved in the cGAS-STING signaling pathway that activates the eukaryotic immune system, effectively suppressing interferon (IFN) production in human cells (26). Different Acb2 homologs exhibit a limited binding spectrum for CDNs and CTNs involved in cGAS-based immunity (9), laying the groundwork for exploring sponge-like proteins that invade eukaryotic cells. The separation of CTNs, such as cA3, could also impact type III CRISPR-Cas immunity due to the shared signaling mechanisms among different immune systems (**Figure 1**) (9).

In summary, sponge-like proteins sequester diverse cOs to antagonize multiple defense systems, creating optimal living conditions for coexistence, with many more sponge-like proteins yet to be discovered.

## 6 Sponge-like proteins provide invaluable insights for overcoming diverse viruses

Sponge-like proteins that sequester second messengers to facilitate complete phage replication and propagation represent a formidable strategy in the interplay between bacteria and phages. In addition to this, other powerful maneuvers are also of significant importance. The reduction of second messengers by nucleases—such as anti-CBASS 1 (Acb1), which has broad cyclic nucleotide hydrolysis activity; anti-Pycsar 1 (Apyc1), which exhibits cyclic NMP phosphodiesterase activity; and anti-CRISPR III 1 (AcrIII-1), which degrades cyclic tetra-adenylate (cA4)—is widely employed by various phages to counteract specific bacterial immune systems (27, 28). Another self-preservation strategy employed by phages is NAD+ reconstitution, wherein phages encode proteins known as NARP1 and NARP2. These proteins restore depleted NAD+ levels by reconstituting NAD+ from its metabolites, thereby curbing cell death induced by Thoeris (29). Furthermore, phage NTases synthesize cOs, competing for recognition of downstream effectors with second messengers. This serves as yet another innovative method for phage replication (30). Nonetheless, the ability of sponge-like proteins to sequester various second messengers, thereby constraining multiple bacterial immune systems simultaneously, is truly remarkable.

Current findings indicate that the sponge-like proteins identified thus far exhibit pronounced hostility toward the CBASS immune system. The eukaryotic cGAS-STING signaling pathway is thought to have evolved from CBASS and retains cGAS-like receptors (cGLRs) that generate second messengers as CD-NTase during evolution. Importantly, the eukaryotic STING pathway, activated by binding to 2’3’-cGAMP, promotes the production of type I interferons that counteract viral infections, while CBASS executes abortive infection through various effectors activated by specific cOs (11, 26). This indicates that evolution has led to more effective and advantageous methods for eliminating threats at a lower cost. Given the similarities between the CBASS and cGAS-STING signaling pathways, alongside evidence suggesting that the defense systems of *Asgard archaea* contribute to the development of eukaryotic innate immune systems, the interactions between bacteria and phages closely resemble those between eukaryotic cells and viruses (31). For instance, Acb1 can degrade second messengers that activate downstream effectors, which is comparable to the action of poxvirus immune nucleases (poxins) that cleave the 2’,3’-cGAMP synthesized by cGAS, thereby inhibiting interferon production (27, 32). Notably, research has shown that poxins can defend against CBASS in bacteria through the expression of chimeric bacteriophages, underscoring the significant interconnections in the long evolutionary history of eukaryotes and prokaryotes (33). Inhibitors of Thoeris and CBASS encoded by phages effectively block eukaryotic immunity, greatly enhancing the likelihood of sponge-like proteins existing in various viruses (24). Research into sponge-like proteins not only highlights their crucial role in these interactions but also suggests broader possibilities for developing antiviral strategies and early prevention methods for infectious diseases.

## 7 Conclusions

The arms race between bacteria and bacteriophages is incredibly intense. Sponge-like proteins represent a novel and powerful weapon that sequesters a variety of second messengers to simultaneously suppress different bacterial immune systems, effectively terminating the process of cell death. This broadens our understanding of the functions of anti-immune proteins encoded by viruses. It is fascinating to explore other sponge-like proteins that play a role in the drastic battles between bacteria and phages, as well as those that may be concealed in viruses that invade eukaryotic cells. Moreover, the intriguing ability of these proteins to bind to cOs while retaining the activity of cOs even after the degradation of sponge-like proteins could pave the way for the development of unique and effective protein tools. Research on sponge-like proteins is ongoing, promising new insights and applications in this field.

## References

[1] Athukoralage JS White MF. Cyclic nucleotide signaling in phage defense and counter-defense. Annu Rev Virol. (2022) 9:451–68. doi: 10.1146/annurev-virology-100120-010228 35567297

[2] Grüschow S Adamson CS White MF. Specificity and sensitivity of an RNA targeting type III CRISPR complex coupled with a NucC endonuclease effector. Nucleic Acids Res. (2021) 49:13122–34. doi: 10.1093/nar/gkab1190 PMC8682760 34871408

[3] Lau RK Ye Q Birkholz EA Berg KR Patel L Mathews IT. Structure and mechanism of a cyclic trinucleotide-activated bacterial endonuclease mediating bacteriophage immunity. Mol Cell. (2020) 77:723–33. doi: 10.1016/j.molcel.2019.12.010 31932164 PMC7065454

[4] Tal N Morehouse BR Millman A Stokar-Avihail A Avraham C Fedorenko T. Cyclic CMP and cyclic UMP mediate bacterial immunity against phages. Cell. (2021) 184:5728–39. doi: 10.1016/j.cell.2021.09.031 34644530 PMC9070634

[5] Ofir G Herbst E Baroz M Cohen D Millman A Doron S. Antiviral activity of bacterial TIR domains via immune signalling molecules. Nature. (2021) 600:116–20. doi: 10.1038/s41586-021-04098-7 34853457

[6] Banh DV Roberts CG Morales-Amador A Berryhill BA Chaudhry W Levin BR. Bacterial cGAS senses a viral RNA to initiate immunity. Nature. (2023) 623:1001–8. doi: 10.1038/s41586-023-06743-9 PMC10686824 37968393

[7] Krüger L Gaskell-Mew L Graham S Shirran S Hertel R White MF. Reversible conjugation of a CBASS nucleotide cyclase regulates bacterial immune response to phage infection. Nat Microbiol. (2024) 9:1579–92. doi: 10.1038/s41564-024-01670-5 PMC11153139 38589469

[8] Roberts CG Fishman CB Banh DV Marraffini LA. A bacterial TIR-based immune system senses viral capsids to initiate defense. bioRxiv: preprint server Biol. (2024). doi: 10.1101/2024.07.29.605636 PMC12578639 41136732

[9] Cao X Xiao Y Huiting E Cao X Li D Ren J. Phage anti-CBASS protein simultaneously sequesters cyclic trinucleotides and dinucleotides. Mol Cell. (2024) 84:375–85. doi: 10.1016/j.molcel.2023.11.026 38103556 PMC11102597

[10] Li D Xiao Y Fedorova I Xiong W Wang Y Liu X. Single phage proteins sequester signals from TIR and cGAS-like enzymes. Nature. (2024) 635:719–27. doi: 10.1038/s41586-024-08122-4 PMC12159963 39478223

[11] Lopatina A Tal N Sorek R. Abortive infection: bacterial suicide as an antiviral immune strategy. Annu Rev Virol. (2020) 7:371–84. doi: 10.1146/annurev-virology-011620-040628 32559405

[12] Rousset F Yirmiya E Nesher S Brandis A Mehlman T Itkin M. A conserved family of immune effectors cleaves cellular ATP upon viral infection. Cell. (2023) 186:3619–31. doi: 10.1016/j.cell.2023.07.020 37595565

[13] Lowey B Whiteley AT Keszei AFA Morehouse BR Mathews IT Antine SP. CBASS immunity uses CARF-related effectors to sense 3’-5’- and 2’-5’-linked cyclic oligonucleotide signals and protect bacteria from phage infection. Cell. (2020) 182:38–49. doi: 10.1016/j.cell.2020.05.019 32544385 PMC7728545

[14] Chang JJ You BJ Tien N Wang YC Yang CS Hou MH. Specific recognition of cyclic oligonucleotides by Cap4 for phage infection. Int J Biol Macromol. (2023) 237:123656. doi: 10.1016/j.ijbiomac.2023.123656 36796558

[15] Fatma S Chakravarti A Zeng X Huang RH. Molecular mechanisms of the CdnG-Cap5 antiphage defense system employing 3’,2’-cGAMP as the second messenger. Nat Commun. (2021) 12:6381. doi: 10.1038/s41467-021-26738-2 34737303 PMC8568899

[16] Rechkoblit O Sciaky D Kreitler DF Buku A Kottur J Aggarwal AK. Activation of CBASS Cap5 endonuclease immune effector by cyclic nucleotides. Nat Struct Mol Biol. (2024) 31:767–76. doi: 10.1038/s41594-024-01220-x PMC11849724 38321146

[17] Severin GB Ramliden MS Hawver LA Wang K Pell ME Kieninger AK. Direct activation of a phospholipase by cyclic GMP-AMP in El Tor Vibrio cholerae. Proc Natl Acad Sci United States America. (2018) 115:E6048–E6055. doi: 10.1073/pnas.1801233115 PMC6042076 29891656

[18] Duncan-Lowey B McNamara-Bordewick NK Tal N Sorek R Kranzusch PJ. Effector-mediated membrane disruption controls cell death in CBASS antiphage defense. Mol Cell. (2021) 81:5039–51. doi: 10.1016/j.molcel.2021.10.020 34784509

[19] Hogrel G Guild A Graham S Rickman H Grüschow S Bertrand Q. Cyclic nucleotide-induced helical structure activates a TIR immune effector. Nature. (2022) 608:808–12. doi: 10.1038/s41586-022-05070-9 35948638

[20] Morehouse BR Yip MCJ Keszei AFA McNamara-Bordewick NK Shao S Kranzusch PJ. Cryo-EM structure of an active bacterial TIR-STING filament complex. Nature. (2022) 608:803–7. doi: 10.1038/s41586-022-04999-1 PMC9402430 35859168

[21] Morehouse BR Govande AA Millman A Keszei AFA Lowey B Ofir G. STING cyclic dinucleotide sensing originated in bacteria. Nature. (2020) 586:429–33. doi: 10.1038/s41586-020-2719-5 PMC7572726 32877915

[22] Tamulaitiene G Sabonis D Sasnauskas G Ruksenaite A Silanskas A Avraham C. Activation of Thoeris antiviral system via SIR2 effector filament assembly. Nature. (2024) 627:431–6. doi: 10.1038/s41586-024-07092-x 38383786

[23] Leavitt A Yirmiya E Amitai G Lu A Garb J Herbst E. Viruses inhibit TIR gcADPR signalling to overcome bacterial defence. Nature. (2022) 611:326–31. doi: 10.1038/s41586-022-05375-9 36174646

[24] Yirmiya E Leavitt A Lu A Ragucci AE Avraham C Osterman I. Phages overcome bacterial immunity via diverse anti-defence proteins. Nature. (2024) 625:352–9. doi: 10.1038/s41586-023-06869-w 37992756

[25] Huiting E Cao X Ren J Athukoralage JS Luo Z Silas S. Bacteriophages inhibit and evade cGAS-like immune function in bacteria. Cell. (2023) 186:864–76. doi: 10.1016/j.cell.2022.12.041 36750095 PMC9975087

[26] Sun L Wu J Du F Chen X Chen ZJ. Cyclic GMP-AMP synthase is a cytosolic DNA sensor that activates the type I interferon pathway. Sci (New York NY). (2013) 339:786–91. doi: 10.1126/science.1232458 PMC3863629 23258413

[27] Hobbs SJ Wein T Lu A Morehouse BR Schnabel J Leavitt A. Phage anti-CBASS and anti-Pycsar nucleases subvert bacterial immunity. Nature. (2022) 605:522–6. doi: 10.1038/s41586-022-04716-y PMC9117128 35395152

[28] Athukoralage JS McMahon SA Zhang C Grüschow S Graham S Krupovic M. An anti-CRISPR viral ring nuclease subverts type III CRISPR immunity. Nature. (2020) 577:572–5. doi: 10.1038/s41586-019-1909-5 PMC6986909 31942067

[29] Osterman I Samra H Rousset F Loseva E Itkin M Malitsky S. Phages reconstitute NAD(+) to counter bacterial immunity. Nature. (2024) 634:1160–7. doi: 10.1038/s41586-024-07986-w 39322677

[30] Ho P Chen Y Biswas S Canfield E Abdolvahabi A Feldman DE. Bacteriophage antidefense genes that neutralize TIR and STING immune responses. Cell Rep. (2023) 42:112305. doi: 10.1016/j.celrep.2023.112305 36952342

[31] Leão P Little ME Appler KE Sahaya D Aguilar-Pine E Currie K. Asgard archaea defense systems and their roles in the origin of eukaryotic immunity. Nat Commun. (2024) 15:6386. doi: 10.1038/s41467-024-50195-2 39085212 PMC11291487

[32] Eaglesham JB Pan Y Kupper TS Kranzusch PJ. Viral and metazoan poxins are cGAMP-specific nucleases that restrict cGAS-STING signalling. Nature. (2019) 566:259–63. doi: 10.1038/s41586-019-0928-6 PMC6640140 30728498

[33] Hobbs SJ Nomburg J Doudna JA Kranzusch PJ. Animal and bacterial viruses share conserved mechanisms of immune evasion. Cell. (2024) 187:5530–9. doi: 10.1016/j.cell.2024.07.057 39197447 PMC11455605

